# “I need them for my autism, but I don’t know why”: Exploring the friendship experiences of autistic children in UK primary schools

**DOI:** 10.1177/23969415241275934

**Published:** 2024-09-05

**Authors:** Laura Fox, Kathryn Asbury

**Affiliations:** 152532 University of York, York, UK

**Keywords:** Autism, friendships, qualitative research, creative methods, special education, gender differences

## Abstract

**Background and aims:**

Autistic children can experience challenges in making and maintaining friendships, and middle childhood (ages 6–12) may be particularly challenging as social networks become more complex. However, a large proportion of research into these experiences is based on adult reports or focuses on the experiences of adolescents, meaning that the voices of younger children are absent. Due to the exclusion of younger children from research, we have a limited understanding of their first-hand experiences of their friendships and the support they receive, which has implications for friendship support and wellbeing. This study aimed to amplify the voices of younger autistic children to explore their first-hand experiences of friendships and highlight areas of social support which may be most beneficial to primary-aged autistic children.

**Methods:**

This study used novel creative methods to support interviews with 19 autistic primary school-aged children to explore their experiences of friendship. Parent-led interviews and scrapbooks supported the children in discussing the challenges and strengths of their friendships.

**Results:**

Children discussed the challenges and strengths of their friendships including the impact of social norms on the need to have friends and their support needs in this area of life. Children also discussed gaps in their current friendships and how they would like to see these filled. It was clear that not all children required or wanted neurotypical-style friendships, with many valuing companionship and gameplay over intimacy. Analysis highlighted the heterogeneity of autistic children's friendships, especially in relation to gender and age, calling for more tailored and individualized support.

**Conclusion and implications:**

Results from the current study show that autistic children can and do have successful friendships but that these friendships may differ from those of their non-autistic peers. The study further adds to the existing literature by showing that younger autistic children can be included in research by using differentiated, accessible and creative methods, and that they are able to voice their opinions on matters surrounding support. It also calls for a tailored approach to supporting autistic children in school and speaking with children to give them autonomy over the support they want to receive.

## Introduction

Friendships play a significant role in children's development, providing a space to practice and learn prosocial behaviors. During middle childhood (6–12 years), friendships develop in complexity, moving from being predominantly based on companionship and play to experiences of intimacy through sharing feelings (Bauminger et al., 2008). As friendships shift from play activities to more complex social interactions, many autistic children report finding friendships more challenging (Cook et al., 2017). This is worrying, as friendships have been found to be protective against challenges that may negatively impact their well-being (Brendgen & Poulin, 2018). As a recent report has highlighted that only 26% of autistic children feel happy at school, and 4 in 5 autistic children have co-existing mental health difficulties (Mon-Williams et al., 2024), understanding autistic children's friendship experiences is of the utmost importance.

Many autistic children have different communication preferences to their non-autistic peers. As a result, there are often mutual misunderstandings which can lead to difficulties with social interactions (Milton, 2012), which can impact children's friendships (Mitchell et al., 2021). Studies suggest that, despite these challenges, autistic children desire friendships, with some experiencing stable friendships (Daniel & Billingsley, 2010). However, the friendships some autistic children desire are not aligned with neurotypical understandings of friendship. For example, many perceive friendship in a qualitatively different manner to their neurotypical peers (Calder et al., 2013), and adult reports often imply a lack of reciprocity in autistic children's friendships (Orsmond et al., 2004). It has been suggested, however, that this lack of reciprocity may be a result of adults having differing definitions of friendships compared to autistic children, who may consider friends to be peers whom they spend time with (Kuo et al., 2013). This suggests that how autistic children's friendships are described and conceptualized may need to be reconsidered. More research is needed into what friendships mean to autistic children if we are to understand how they can be best supported. This is especially important in primary-aged children when the move from playmate activities to more complex intimate relationships is developing (Bauminger et al., 2008).

Although research into autistic children's friendship exists, the current literature base is predominantly quantitative, reporting on friendship characteristic which are measurable, such as number of friends, peer nominations, or frequency of interactions (Petrina et al., 2014). It is also common for studies to focus on the perspective of parents and teachers (Rowley et al., 2012), or explore the experiences of adolescents or young people (Finke, 2023). While these studies are important in exploring autistic children's friendships, it may result in a fragmented or biased understanding of this phenomenon. For example, most autistic adolescents attend secondary school settings, which often provide more diverse opportunities for interacting with peers which will likely impact on their experiences of friendships (Jindal-Snape et al., 2020).

Furthermore, the voices of autistic girls are underrepresented in the friendship literature, which is not surprising given the higher prevalence of males diagnosed with autism (APA, 2013). However, this has implications for understanding friendship development in autistic individuals. Given the gender differences in social norms and friendships that are evident in neurotypical children, with girls often emphasizing emotional closeness and engaging in more communication-based activities than boys (Dean et al., 2014), it is likely that the expectations for autistic girls to have friends that follow these norms will be gendered, something which may be challenging for autistic individuals. Recent studies have highlighted key gender differences in autistic children's friendships, with girls reporting more relational and overall conflict compared to autistic boys and non-autistic peers (Sedgewick et al., 2019), and many favoring companionship over emotional closeness (Cook et al., 2017). This may suggest that the gendered norms of neurotypical friendships are being placed on autistic girls, resulting in conflict, as opposed to individuals being able to be supported in developing and maintaining friendships in a way that is best suited to them. Therefore, autistic girls may require different forms of support compared to boys, and interventions will need to be tailored to their needs given that their responses to certain elements of friendships, such as emotional connectedness, may be socialized as opposed to an intrinsic response. However, very little research focuses on the experiences of younger autistic children, with recent work focusing on the experiences of adolescents (Halsall et al., 2021; Myles et al., 2019). Therefore, qualitative studies with younger autistic children that includes young autistic girls can help build upon these findings to gain an in-depth picture of how they feel about their friendships and the support they may want to receive.

Throughout the literature, there is an absence of the voices of younger autistic voices. One reason for this may be that working with children who may have non-typical communication skills comes with challenges (Ellis, 2016). However, these may be mitigated by using more creative methods. Drawing has been found to offer a non-verbal mode of expression for children, placing the child in the position of being the expert (Leitch, 2008). Furthermore, drawings and written data have been used successfully to support discussions with children with social communication challenges (Hambly, 2014). Including flexible methods will allow more younger autistic children to participate in research, which will, in turn, help us better understand their friendship experiences directly, as opposed to relying on quantitative measures or the reports of key adults or older autistic young people. Doing so will help to guide policy and the design of support, helping to ensure interventions are designed appropriately for autistic pupils in this age range to support friendship development and maintenance better.

Therefore, this study collected data from primary-aged autistic children using creative methods to explore their first-hand experiences of friendships to address the following research questions:

- How do autistic children attending a primary setting in the UK experience their friendships?
- Are there age and gender differences in autistic children's friendship experiences?

## Methods

### Participants

Participants were 19 children aged 7–11 years attending a UK mainstream or special primary school. Children were required to have an Education and Health Care Plan (EHCP), which is a legally binding document that outlines their support needs and formal diagnosis. They were recruited via social media, support groups and primary schools. Information on participants can be found in Table 1.

**Table 1.** Child demographic information.

Pseudonym	Age (years)	Gender	School setting
Kursi	7	F	Mainstream (MS)
Benjy	8	F	MS
J	9	F	MS
Bella	9	F	MS
Aahh	9	F	MS
Yaya	10	F	MS
FreyFrey	10	F	MS
Newton	11	F	MS
Sandie	11	F	MS
Hugo	7	M	MS
H	8	M	MS
Lennie	8	M	MS
Loodoluf	8	M	MS
RNHC	10	M	MS
Eli	11	M	MS
W	9	M	Special School (SS)
Cody	9	M	SS
Morgan	10	M	SS
Adam	11	M	SS

### Materials

#### Scrapbooks

**Figure 1.**
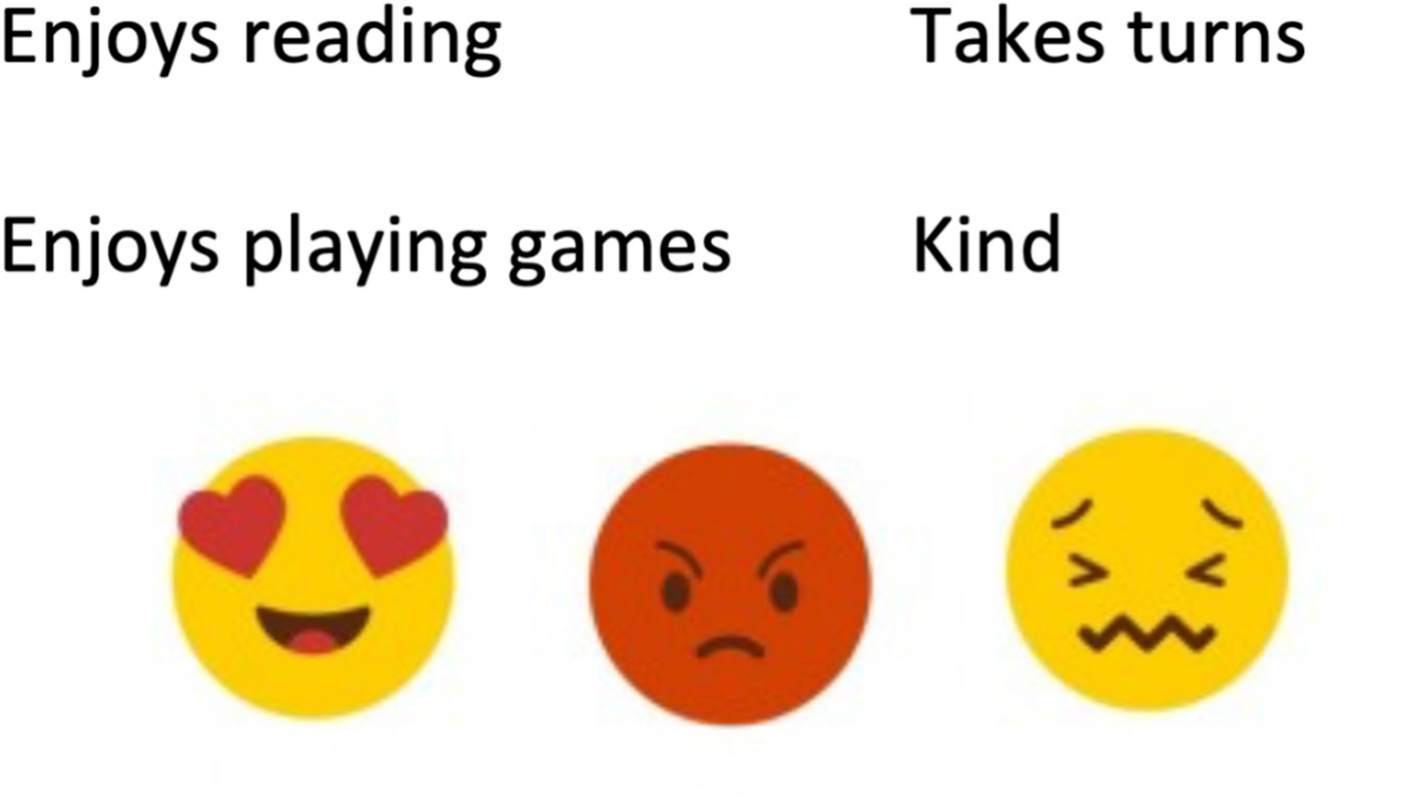
Example of keywords and emojis.

Scrapbooks facilitated parent-led interviews, allowing children to describe their experiences through drawing, crafts, and writing. Tasks explored children's ideas of a perfect friend and their experiences with real-life friends. Emojis, playdough and keywords were provided to support activities (Figure 1). Children were asked to complete the scrapbook independently, but prompts were provided for parents to use if needed.

The scrapbook was designed by the first author and structured around the main research questions. The idea to use a scrapbook activity was based on prior successful use of the method with children with learning difficulties (e.g., Beresford et al., 2004; Ellis, 2016; Hambly, 2014), and through the first authors experience of working with autistic children. The activities in the book were reviewed by a colleague with Early Year Foundation Stage SENDCo experience, and a Year 4 mainstream classroom teacher, to ensure that the level was appropriate for the age range. The activities were then piloted with a 9-year-old boy who receives speech-language therapy and was awaiting an autistic assessment. The choice to use a child who was not formally diagnosed with autism was made so that an eligible child would not be excluded from the main study due to piloting. Piloting resulted in the removal of some emojis and labels which were similar in meaning from the pack to reduce the number of choices on offer at the suggestion of this child's parent.

#### Parent-led interviews

Data collection was completed during COVID-19 lockdowns, meaning that parent-led, remote interviews allowed data to be collected in a COVID-safe way and at a time convenient to participants. They also allowed children to be in a safe space with a family member with whom they may have been more comfortable talking about a possibly challenging topic than with an unknown researcher, thus reducing the power dynamic which is often prominent in qualitative research, and may have allowed children to express themselves more freely (Punch, 2002).

All materials can be found on the link anonymized.

### Procedure

Ethical approval was sought through the ethics committee. Parents/carers were provided with online information sheets and provided informed written consent and a postal address for packs to be sent to. Children received a child-specific information sheet (see Appendix A). This explained to children what research was, and what would happen with their data. After reading through the information sheet, children were asked to provide their assent via an information page at the beginning of the scrapbook before participating in the study. Parents were provided with a semi-structured interview script related to the activities in the scrapbook and were asked to video- or audio-record their children talking with them. As there was a chance that parent-led interviews may lead to bias, with parents potentially influencing children's responses, parents were encouraged to ask the questions in as similar a way as possible to those on the interview schedule but were reminded that if their child was unhappy talking about certain tasks, that the question could be skipped. Parents were informed that they did not have to discuss each task in order or in a single sitting, but that they should make sure that all discussions were recorded, even if this meant that the interview came through as multiple video recordings. It was hoped that by doing so, any potential bias would be reduced. Recordings were returned to the researcher via a secure drop-off link. Scrapbooks were returned via post, or photographs were uploaded via the link if children wished to keep their work.

### Positionality

It must be acknowledged that there are factors that may influence the interpretation of the data. Firstly, this work has been carried out through a critical realism lens, where the authors acknowledge that individuals conceptualize different perspectives and representations of a singular reality and that, as researchers, we must interpret these experiences the further understand the phenomena being explored (Archer et al., 2013). Furthermore, the first author has experience working with autistic children and the second author is parent to an autistic child, which may have made them particularly aware of the challenges some children face. Furthermore, the authors’ understanding of autism is influenced by the neurodiversity movement in which autism is viewed as a natural and valuable part of human variation, which may influence analysis (Bagatell, 2010; Kapp et al., 2013). Despite this, every effort was made to ensure the data were represented through the lens of the participants by engaging in reflexivity in the form of a research diary and through peer discussions.

### Analysis

Participants’ accounts were analyzed by the first author using reflexive thematic analysis (RTA), an interpretative, six-phase approach, which aims to identify and analyze patterns in a dataset (Braun & Clarke, 2021).

1. Transcripts were read and re-read and notes relating to analytic ideas or observations were made.
2. Codes were developed by systematically working through the dataset, with segments of data thought to be relevant and meaningful being given code labels.
3. Code labels were then collated, and relevant segments of data were compiled for each code.
4. These codes were then checked with the second author to ensure there were no overlaps.
5. Initial themes were created by compiling codes which shared core ideas. Themes were then reviewed and refined, ensuring that they were built around a strong core concept, before being named. The final analysis was once again shared with the second author before any final refinements were made.
6. The analysis was written up to present a coherent story about the dataset.

The analysis for the study was pre-registered. Scrapbooks were not analyzed on this occasion as they were intended as a springboard for the interview rather than a form of data for analysis. However, excerpts are used to contextualize and illustrate interview data.

## Results

**Figure 2.**
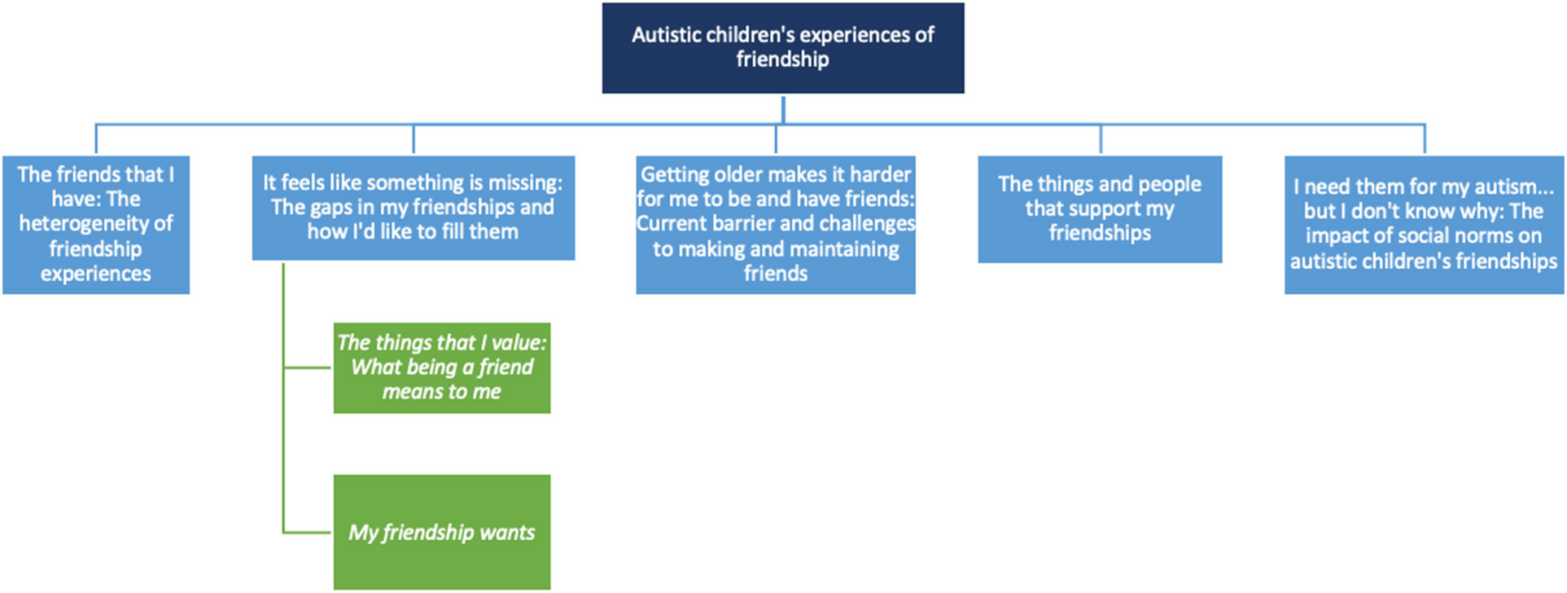
Thematic map of children's friendship experiences.

Five themes were constructed from the data, which are presented in Figure 2.

### The friends that I have; the heterogeneity of friendship experiences

Children spoke about real-life friends, with some having an abundance of successful friendships and others reporting none. Children placed differing levels of importance on friendships, acknowledging that having friends was important to being happy.

**Figure 3.**
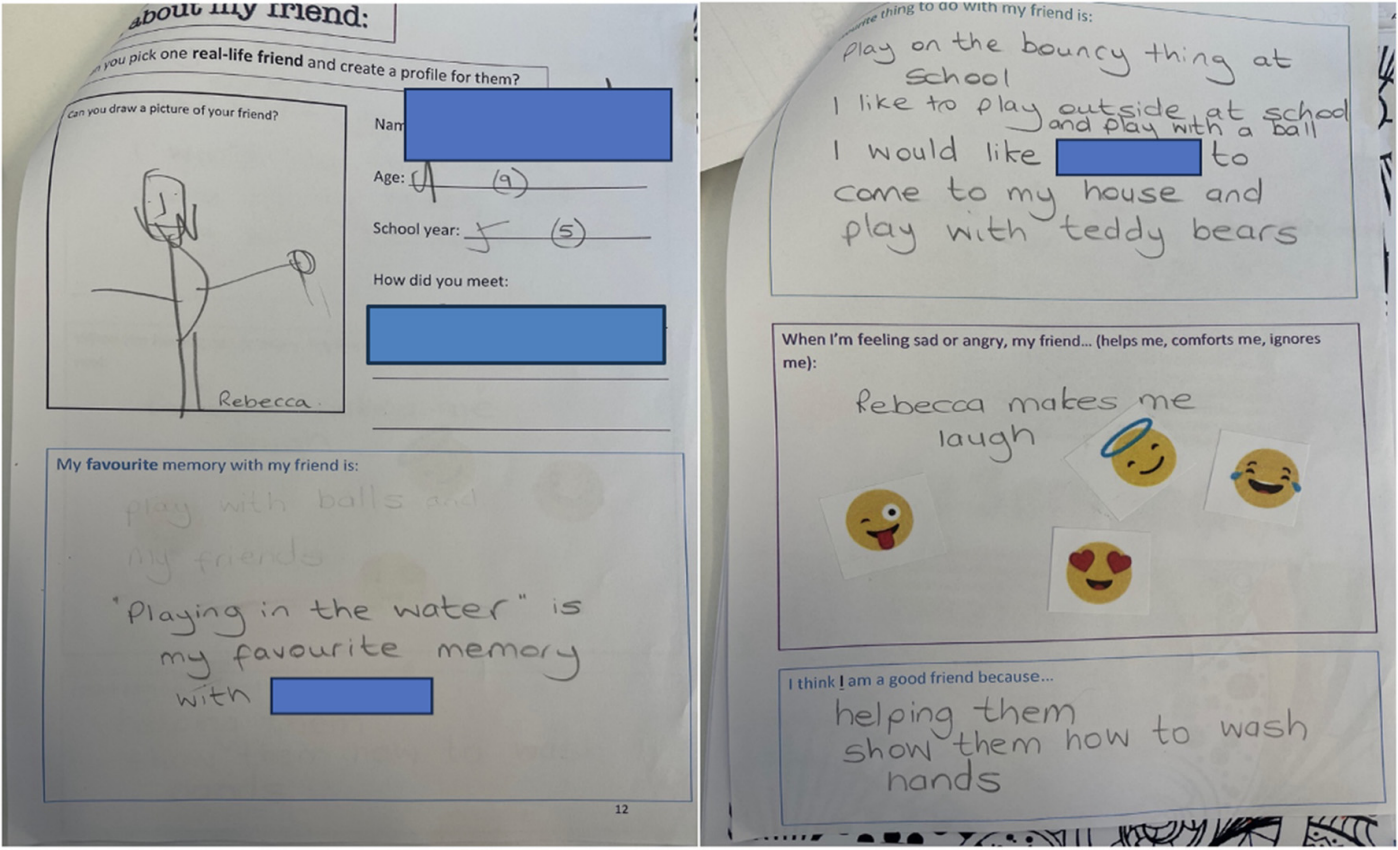
Scrapbook extract from J (F, 9, MS) of their real-life friend.

Many expressed that they had successful friendships, with some having a good group of friends at school. Others specified having a best friend: “[name] […] she's my best friend at school” (Benjy, F, 8, MS) and many had friendships that lasted over time, with some maintaining friendships since reception (age 4–5 years) (Figure 3):

P: Where did you meet [name]? How did you meet her? Can you remember?

C: Er [place] reception

P: You met her in reception […] So you’ve known [name] a long time haven’t you

C: Yes (J, F, 9, M)

Although many spoke of successful friendships, not all children showed an interest in peers. Several boys, but not girls, were ambivalent towards friendships and did not want to seek new friends actively: “P: So if those three people weren’t in, would you try and make new friends or would you play on your own? C: I’d play on my own” (Loodoluf, M, 8, MS).

**Figure 4.**
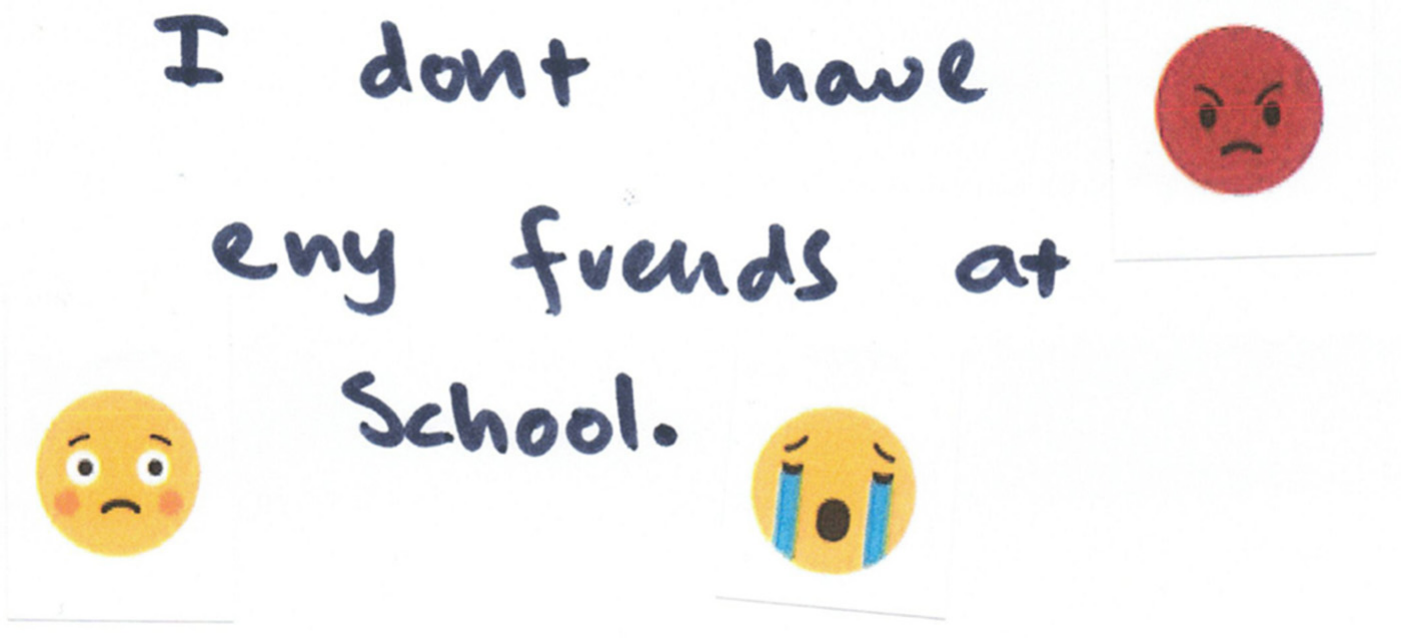
Scrapbook extract from YaYa (F, 10, MS) showing her lack of friends.

For some, however, this lack of friends was reported to result in feelings of loneliness: “I have no one at school, so I have no one to play with so I just feel lonely and left out” (YaYa, F, 10, MS) (Figure 4).

**Figure 5.**
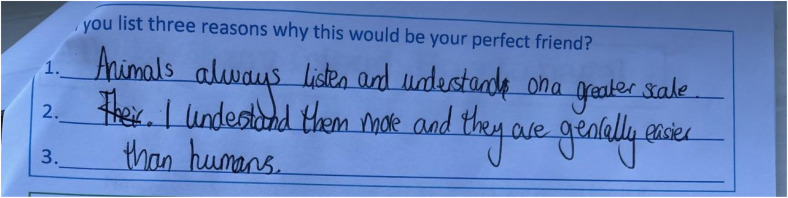
Scrapbook extracts from Newton (F, 11, MS).

Some children overcame this loneliness by finding friendships in animals: “Because I think [name] […] and [name] are my most set solid friends” (Newton, F, 11, MS) (Figure 5).

A small number of children reported that they were unsure about their current friendship status. Although children named people in their class that they spent time with, some were unsure if this was a friendship: “[I] can barely even tell if we are friends or just two people that go to the same school” (Newton, F, 11, MS).

Despite the differing levels of friendships across the group, many noted that having friendships was important and ascribed value to successful friendships. Children discussed how friendships offered sources of support, with some being aware of the benefits to well-being: “It's good for mental well-being or mental health and just, yeah I think people should have friends” (Newton, F, 11, MS). Others were attuned to friendships helping with loneliness: “Yeah, it matters because it's like someone who's, who like cares about you a lot and you’re like, not alone” (RNHC, M, 10, MS). This suggests that although many children encountered challenges with their friendships, they still ascribed value to having and being a friend and were conscious of the positive benefits that may come with friendships.

### Feels like something is missing: the gaps in my friendships and how I’d like to fill them

#### The things that I value: what being a friend means to me

The things children valued in their friendships differed, with some emphasizing the need for kindness and others stating that having fun was the priority. Children across genders and ages spoke of how being kind was important. For some, real-life friends showed kindness during challenging times at school:

This is [name] he asks me if I’m ok, he says ‘have you told anyone?’ and if I say no he says ‘oh yeah how about we tell a grown-up’ and then he's just really nice to me when I’m sad (Loodoluf, M, 8, MS)

**Figure 6.**
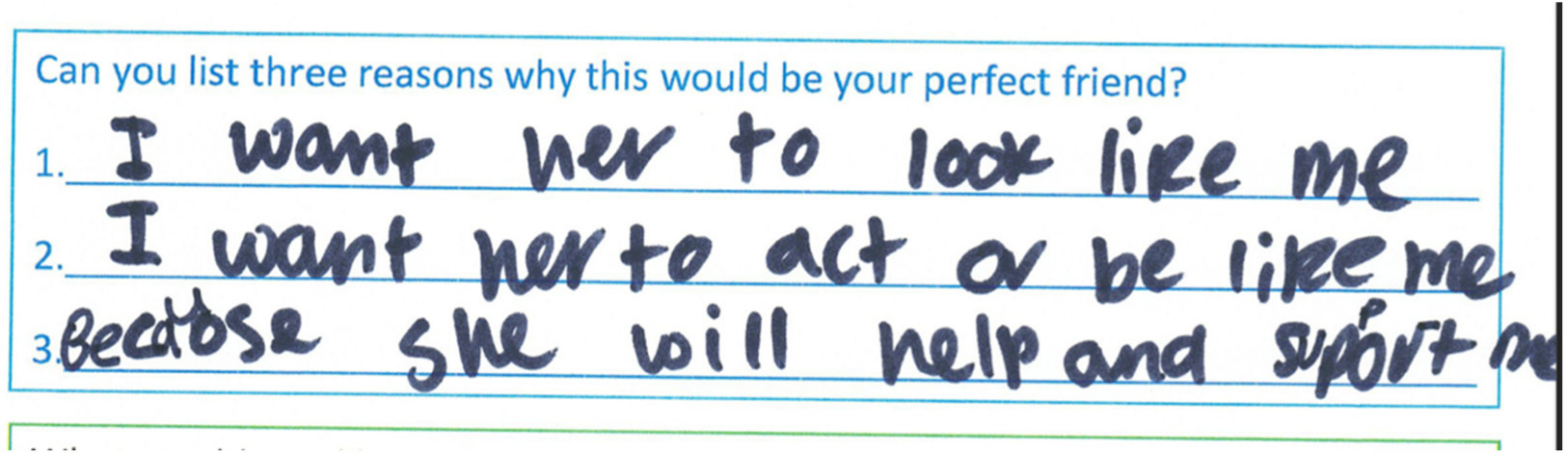
Scrapbook extract from YaYa (F, 10, MS) showing her friendship wants.

**Figure 7.**
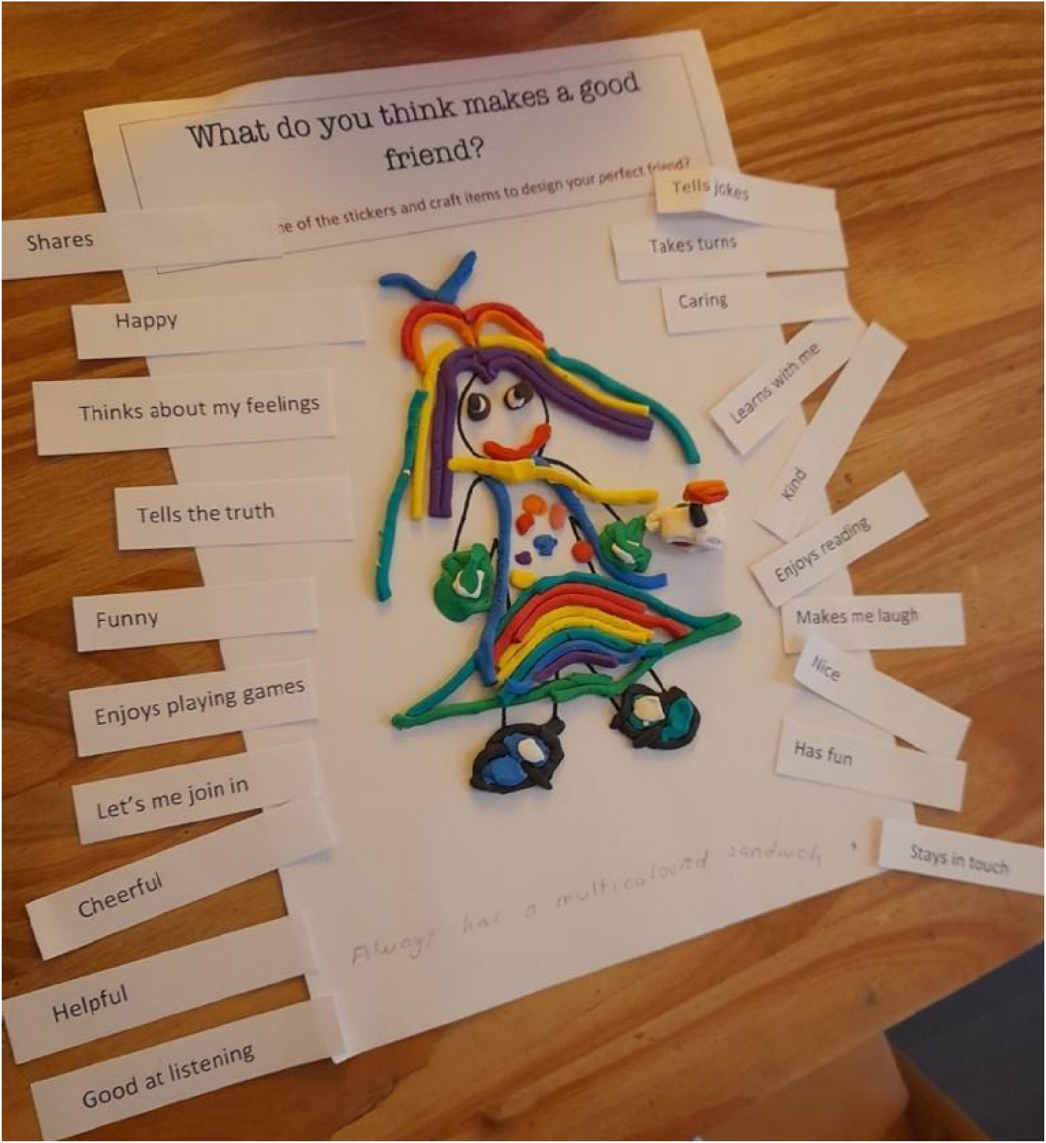
Scrapbook extract Kursi (F, 7, MS) showing a good friend.

**Figure 8.**
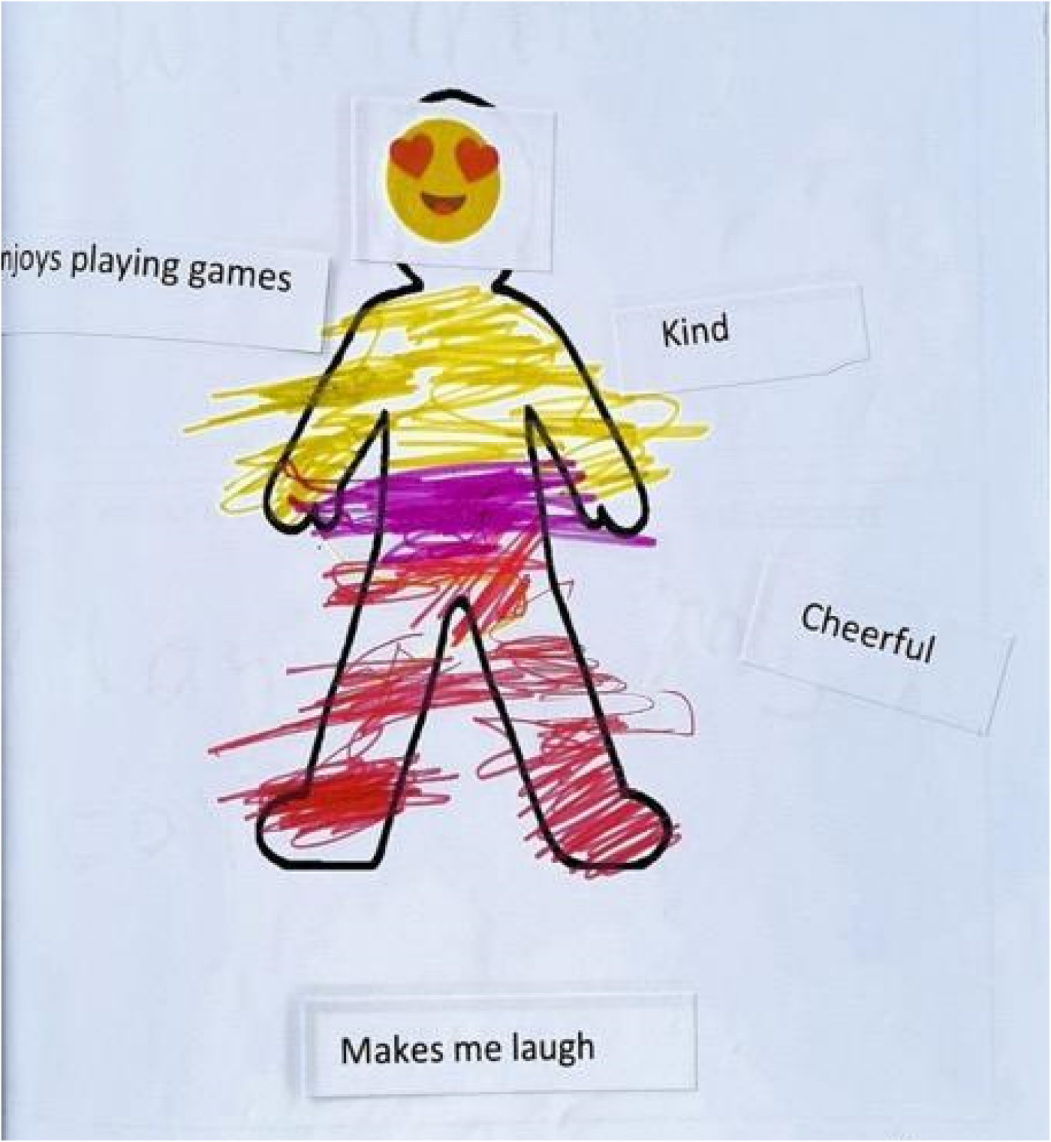
Scrapbook extract from W (M, 9, SS) showing the values he believed were important in a friend.

Others reflected on how their ideal friend would be understanding of their feelings: “Someone who is always, thinks about my feelings because they, they won’t hurt my feelings and sometimes be mean to me, they’ll always be fun and nice” (YaYa, F, 10, MS) and wanting help and support was evident in YaYa's scrapbook in Figure 6 (Figures 7 and 8).

Alongside being kind, many discussed the need for friends to make things fun: “I like people who tell jokes and are funny because they, it's just they’re very, my type of friend because it's not someone who's always serious, someone who's a bit more like silly” (YaYa, F, 10, MS) and having fun appeared to be a key motivation for spending time with friends.

Children discussed the number of friends they believed would benefit them. For some, a smaller number of friends was valued: “It would be nice with one friend, it is nice with one friend” (Bella, F, 8, MS). However, others believed larger groups would be more fun: “Because the more people there is, the more fun it is […] It's just like, that's how it works” (Benjy, F, 8, MS). It is therefore likely that autistic children will show different preferences for not only the type of friends they want, but also for the number of friends.

#### My friendship wants

Children spoke of how having friends that would play with them was something they would enjoy but were lacking. Interestingly, despite previously stating that they would be happier to play alone, some children eluded that this is not always the case: “Because I don’t wanna play on my own all the time, I do it sometimes, but I want to play with other people” (Loodoluf, M, 8, MS). It was clear that children wanted friends who let them join in and were inclusive but this might change depending on time and context.

The types of games that children wanted to play were closer to the preferences of younger children, such as role-play or structured gameplay, and this was something children spoke of being missing from their current friendships:

P: If your friend came home with you, what would you do after school?

C: Play lego and teddy bears” (J, F, 9, MS),

Finally, some expressed the want for more emotional support: “Because they can help me when I get emotional” (Benjy, F, 8, MS). Others said they would like friends who listened and supported them: “I don’t know why, it's just another person I can rely on […] she would listen to me” (Aahh, F, 9, MS). Children across the study valued different aspects of friendships, highlighting the challenge with one-size-fits-all support. Differences were evident not just between children, but within children, as highlighted by Loodoluf's changing wants.

### Getting older makes it harder for me to be and have friends: current barriers and challenges to making and maintaining friendships

**Figure 9.**
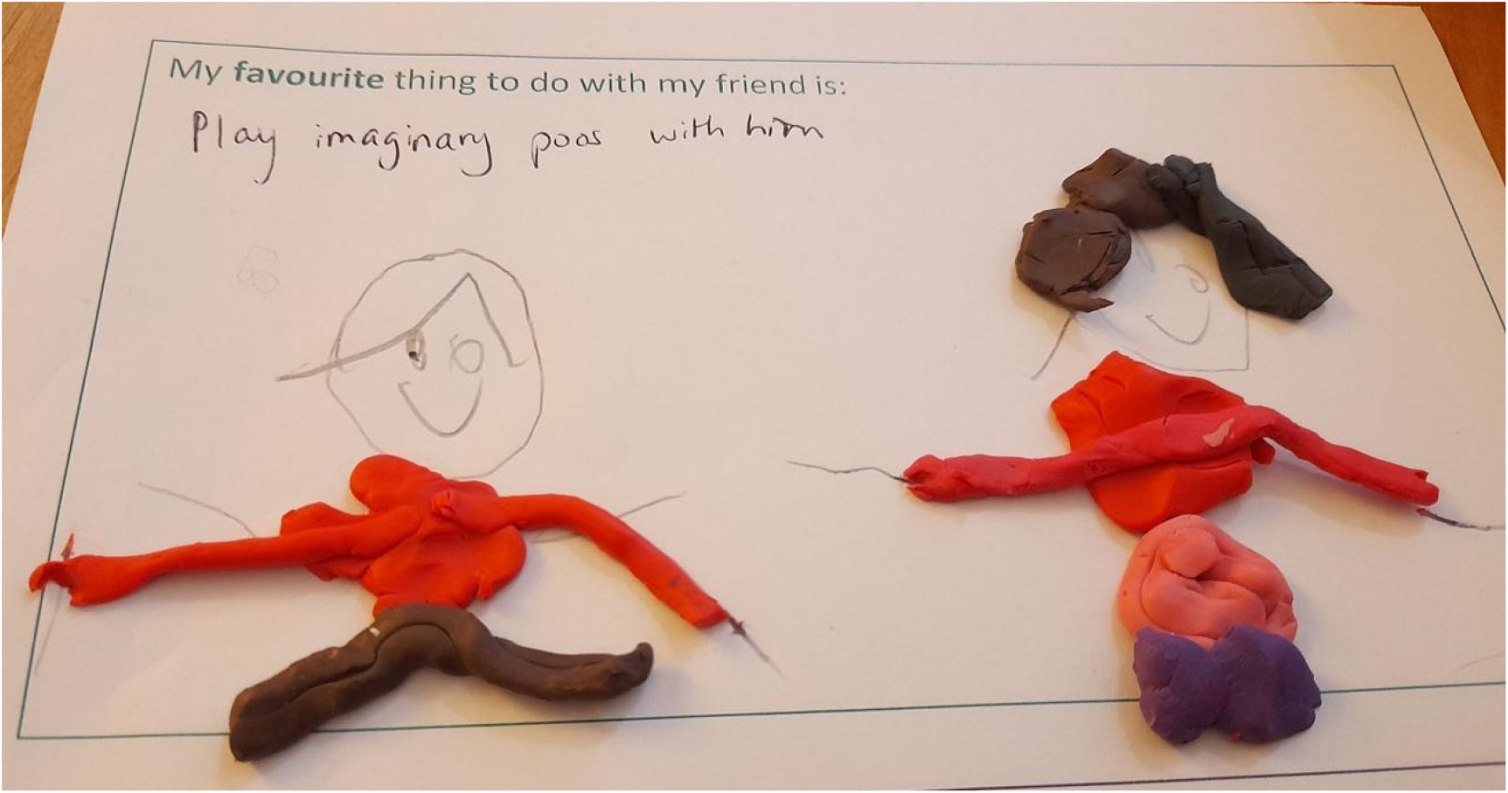
Scrapbook extract Kursi (F, 7, MS) describing her favorite gameplay activity.

Children spoke of the difficulties they experienced with current friends and how these had worsened over time. Children described how peers’ play preferences were maturing and that a social gap was becoming more evident as they moved into the later years of primary school. Many reported that they would prefer to engage in gameplay over talking or doing more “grown-up” activities with peers (Figure 9):

P: OK. Can you tell me what you’re doing with this friend at school?

C: Playing together

P: Why did you pick that?

C: Because it's fun, and if we play together he’ll be a perfect friend to have (Lennie, M, 8, MS).

As children moved through primary school, this change in play preferences resulted in a loss of friends, and many highlighted that a significant barrier to engaging with peers was the shift towards conversation-based activities:

It's hard to make friends because in year two there was this new person who came and I wanted to make friends with her but I was too scared, I didn’t know what to say to her and, I was trying to say something, but everyone was laughing at me because I was too scared to talk to her and I got scared because she was looking at me and I didn’t know what to say to her. (YaYa, F, 10, MS)

Across genders, children expressed how conversations were easier with younger friends: “You can’t just be like ‘Hey can you be my friend?’ they would be like ‘ehhhh?’ that would have been much easier if that was in like nursery” (RNHC, M, 10, MS). Furthermore, some felt that when talking to peers, this was often done in the wrong way, suggesting that some children see neurotypical forms of communication and friendship as “correct”:

I started to lose my friends because I would talk to them sometimes, but I would say it in the wrong way and then they would get sad and upset because I was being mean to them and then they didn’t want to be my friend anymore because I kept messing up when I talked to them (YaYa, F, 10, MS)

For those who had developed friendships, many experienced difficulties with maintaining them and feeling excluded:

It's always who's whoever comes out last or whoever joins the game last has to be the stucker and I’m always the one who joins the game last or is the last one outside because I’m slower so then I have to always be the stucker and it's unfair (YaYa, F, 10, MS)

This experience of exclusion was spoken about by many, and it was evident exclusions were taken personally by some: “Because [name] always abandoned me when I asked her to do it with me” (Sandie, F, 11, MS).

For others, friends moving away made maintaining friendships challenging:

So me and [name] used to be good friends, we are still friends we just used to be together a lot, like five years ago or six years ago but after he moved to [place] I rarely see him and even worse the pandemic is putting us in a worse, worse, worse position (RNHC, M, 10, MS)

This loss of friendships due to physical distance was spoken about by older children who expressed concern over school transition and the possibility of not moving with their current friends. These children spoke of how transitioning without current friends would mean they may need to make new ones, something which some children expressed was not necessarily their first choice: “I feel, it feels sad to move on but we got to move on” (RNHC, M, 10, MS).

### The things and people that support my friendships

Children discussed the things and people that helped support their friendships, including support mechanisms such as technology and support from parents and school. Parental support was mentioned by some girls who reported that parents helped by organizing playdates: “(nods) and you invite her to my house” (J, F, 9, MS).

For others, school played a supportive role, providing a place to meet and make friends with many speaking about how existing friendships had started at school: “P: Can you remember where you met your real-life friends? C: In school” (Loodoluf, M, 8, MS). School was also seen as a familiar setting which provided a safe place to talk to peers. Interestingly, although school as a physical place was spoken about, children did not explicitly speak about how adults in school supported them. However, it was clear that many had difficulties at school and wanted more help, suggesting they did not feel supported sufficiently:

P: But if you went to a different school with children similar to you and maybe if you had adults there to support that friendship, to help you, would that help you? Would that make you happy about making friends?

C: Yeah

P: And wanting friends again

C: Probably (YaYa, F, 10, MS)

Alongside this, having shared interests was supportive. For some, having a shared interest they could use as an anchor point for conversations was helpful, and children often included their favorite activity or object as their favorite thing to do with friends:

P: Ah brilliant, why do you think liking dogs is important to you?

C: So they can come to my house and my play with my dog […]

P: So what would you like to do when you’re playing at school with your perfect friend?

C: Chat about dogs (Benjy, F, 8, MS).

Others found the types of play they engaged with facilitated friendships. As mentioned earlier, many preferred active play or structured play: “I find it easier to have structured play” (Newton, F, 11, MS).

The role of technology was discussed by many. Some found messaging apps or video calls a successful way of communicating with existing friends. For others, online gaming was a way they could build new friendships, as well as using gaming as a platform to maintain current friendships: “If it's a game where there's only one player, I have to play it by myself but they can watch but if it's multiple players I like to play with them yeah” (Aahh, F, 9, MS).

### I need them for my autism… But I don’t know why: the impact of social norms on autistic children's friendships

Children discussed how being aware of their diagnosis made them feel different from their peers and the impact of masking on friendships. Girls, but not boys, spoke about feeling the need to have friends because of their diagnosis but were unable to explain in more detail why they believed this was important:

P: Do you think it's important that you have friends?

C: Yeah, especially for my autism yeah

P: Go on then, tell me more

C: I can’t really explain more, sorry (Aaah, F, 9, MS)

The influence of neurotypical norms was also seen in how some discussed the “rules” surrounding friendships, such as not being mean or needing to be nice, which impacted their interactions with peers. This was often done by mimicking how neurotypical children engage with friends, with some being aware that certain rules must be followed simply “because that's what friends do” (RNHC, M, 10, MS). Furthermore, older children spoke of how they changed behaviors to fit in with and make friends with their peers: “I think it was my idea, I think I had a lot of energy then so I was trying to fit in” (Newton, F, 11, MS). However, taking part in games that others were playing to avoid being left out was often at a cost to their happiness:

It made me feel sad because I still wanted to play those games, I didn’t, I didn’t get to play those games anymore, I had to play like, their games but I didn’t want to play that because I didn’t, I didn’t want to play by myself (YaYa, F, 10, MS)

The examples show how masking is used to navigate social spaces and hide challenges. Masking may have consequences for children who hide their difficulties to the point of not receiving support. For example, children spoke of how engaging in play impacted the way adults interpreted friendships: “I don’t know if they know that, well we had this PSHE thing at school and I said ‘I have no friends’ and then the teacher said ‘of course you have friends, you play with these people every day’” (YaYa, F, 10, MS). It may be that by masking friendship difficulties and playing with others, autistic children miss out on support and are, therefore, unable to develop further friendships that better meet their needs.

## Discussion

This study explored the experiences of primary-aged autistic children in relation to their friendships. Past literature has highlighted the challenges some autistic children face with making and maintaining friendships, with some suggesting social differences may lead them to be perceived as wanting to be on their own or prefer solitary play (Portway & Johnson, 2003). The participants here painted a different picture, and it was clear that many, but not all, had a want for friendships, and that autistic children can and do have friendships that they deem valuable. However, the things children wanted from their friendships varied, with some calling for companionship and others expressing a want for emotional connection. Despite these differences, all children showed a want for gameplay, in line with previous literature (Daniel & Billingsley, 2010), and understanding that playing with others is a valued part of friendship for autistic children needs to be a priority to key adults.

### Age differences

Although past literature has suggested that autistic children may have difficulties maintaining friendships, some children in this study reported not only having successful friendships, but having a best friend and friendships that lasted over time. A key finding from this study was that children's definitions of what a friend is differed from those of neurotypical friendship expectations, especially as children grew older, which has implications for support.

In line with previous literature, many children in this study showed a preference for structured gameplay and a large proportion of children reported a want for companionship over emotional ties, which may explain some children's preference for playmates rather than soulmates (Calder et al., 2013). It is thought that this may be linked to autistic children's need to have varied sensory elements in their play, such as running and jumping freely, as opposed to following active play with set rules such as football, features of play that are more commonly found among younger children (Fahy et al., 2020). As preferences align more with younger neurotypical friendships, children here reported a widening social gap as they moved towards the end of primary school.

It could be suggested that these changing friendship experiences are connected with developmental processes, such as the development of social and communication skills (Durkin & Conti-Ramsden, 2007). As non-autistic children develop more complex skills, their preferences for spending time talking may become a priority; it was clear that challenges increased for many autistic children with age and older children reported being more aware of their differences and felt more pressure to conform to social norms. This could suggest that children in the final few years of primary school may be at higher risk of friendship break downs and may benefit from having the opportunity to engage in gameplay with children who show similar interests or children who are younger in age.

Although many discussed a preference for companionship, especially those that were younger, it is important to note that some children did discuss a want for emotional connections with peers which goes against previous findings. In a recent systematic review, Fox et al. (2024) found autistic children showed a preference for companionship over the sharing of emotions. However, many studies in the systematic review did not focus directly on children's experiences, using adult reports or observations instead. Here, however, older children expressed a desire for a level of emotional connection with friends and spoke of how they were attuned to friendships being important for loneliness and good mental well-being. This suggests two things, firstly, adults may not accurately interpret what children show a preference for in their friendships. Secondly, older autistic children may need and want different things from their friendship. This shows that it is crucial for children to be asked about their friendship wants and experiences in accessible ways in order to deepen our understanding of friendships and, in turn, design more appropriate support.

Children across the study also spoke of how they engaged in masking to limit friendship challenges. In line with previous research, masking behaviors were more commonly reported by older children (Ross et al., 2023). Studies have shown that as children move towards adolescence, individuals begin to mask interests that may be seen as immature. For example, Halsall et al. (2021) found that girls struggled with their desire to participate in “younger” games while recognizing that their neurotypical peers had progressed to more “grown-up” activities, thus hiding their play preferences. This is reflected throughout the experiences of the children within this study.

Although masking is often used as a coping mechanism in social situations (Tubio-Fungueirino et al., 2021), and has been shown to be protective and harmful for autistic children (Sedgewick et al., 2021), it also has links to mental health difficulties (Chapman et al., 2022) and negative impacts on identity development (Berkovits et al., 2020). Children in this study who spoke of masking behaviors were all attending mainstream schools. It may be that the need to mask is more prominent within mainstream settings, given that children are surrounded by more neurotypical peers as opposed to those in special school who would likely interact with other autistic individuals or children with learning disabilities. Furthermore, children with greater language abilities have been shown to mask more often as they have the skills to imitate and mimic others (Happe, 1995). Given that children in mainstream education may have higher language skills, even those with an EHCP, this may also be another explanation for the prevalence of masking in this study.

Moreover, in line with the double empathy problem, research highlights the benefits of socializing with other autistic individuals, as there is no need to mask social behaviors if an individual's preferred method of communication is accepted (Crompton et al., 2020). This suggests that mainstream schools may benefit from providing autistic children with spaces to socialize with other neurodivergent individuals. Mainstream schools may also benefit from promoting an understanding social communication as two-way interactions. For example, when autistic people are not understood using their own communication styles, they often feel that they need to comply with neurotypical norms (Milton, 2012). Providing support for all children on how to communicate successfully with others may help to support not only friendship development but allow autistic children to be accepted for who they are, which may improve quality of life and well-being (Cage et al., 2018).

It was also clear that, for older children, transition was a particular time of concern in regards to their friendships. School transitions are especially challenging for autistic children (Hannah & Topping, 2012). Some may move schools with their friends; others may move at unconventional times due to support needs or to a special school outside their neighborhood. As friendships can be protective during the move to a new school (Ng-Knight et al., 2019), understanding autistic children's friendship wants may be vital for supporting these friendships during and after transition. Given the differing transitions autistic children may experience not only from their neurotypical peers, but from other autistic children (Fox et al., 2023), speaking with children about their concerns and support needs may reduce feelings of anxiety and may not only support successful transition, but also support friendship maintenance across settings.

### The influence of gendered norms

There are differences in the experiences of children within this study, with girls facing differing social pressures than boys, including the expectation to have a close-knit group of friends, even if this is not what they desire. Girls reported placing more of an emphasis on wanting to fit in with their peers, often resulting in an increased use of masking. Studies have shown that adolescent girls report masking behaviors to be a coping mechanism which is often used to aid with friendship development (Tomlinson et al., 2021). As discussed above, this can be at the expense of autistic individuals’ mental health (van der Putten et al., 2023), and therefore supporting girls in not feeling the need to mask consistently at school may be beneficial to their well-being in the longer term. This may mean that more needs to be done to educate autistic individuals on gendered norms (e.g., emotional connectedness) to make sure that they can make decisions about their friendships that are intrinsically motivated and not based on social pressures. A recent project by Powell et al. (2024) has found that co-designed activity booklets are an accessible way of educating autistic children and young people about their autism, and activities based on this approach with a larger emphasis on friendships may be one way to support autistic individuals in this area.

Furthermore, girls appeared to be more concerned about their friendships than boys. This is not to say that boys were not motivated to make friends, but they appeared to attach less pressure to making and keeping friends than girls did. Girls expressed that they felt the need to have friends and spoke about social norms more often than boys. This finding is corroborated by past research which highlights how autistic adolescent girls were more concerned with and placed more value on neurotypical friendship norms, while boys had less motivation and were less concerned with the need to make and keep friends (Sedgewick et al., 2016, 2019). This shows that younger autistic girls may feel just as much pressure as their adolescent peers to conform to social norms and autistic girls may require additional friendship support throughout primary school to ensure that their well-being is protected.

### Areas of success

Despite the challenges above, many children reported that shared interests and structured gameplay did support their friendships. Studies have shown that having a shared interest or a common activity is a key requirement and marker of autistic individuals’ friendships (O’Hagan & Hebron, 2016), and links between having a shared interest and a sense of belonging have been identified (Sosnowy et al., 2019). Understanding that having structured tasks and shared interests is supportive has implications for interventions and support at home and school. Support plans could include opportunities to engage with peers in a structured way, which is likely more of a challenge as children age, and providing children with spaces to engage with shared interests, such as games club or after-school clubs, could be a way that schools can support autistic children. Furthermore, a preference for internet communication/gaming was reported by children across the study, and it has been suggested that engaging with gaming/messaging may help remove the stress of face-to-face interaction while incorporating a shared interest (O’Hagan & Hebron, 2016; Ryan et al., 2020). More information on navigating online spaces for parents and school staff may be one way this preference for online connections could be supported which may in turn support successful friendships.

Finally, children in the study identified school as a safe space to make and engage with friends, however, there was distinct lack of discussion surrounding explicit support that schools offered. Understanding that children identify school as a place in which they would be comfortable initiating interactions with friends shows that school-based interventions, based on children's preference for support, may be highly successful in helping autistic children to make and maintain friends.

### Strengths and limitations

The use of creative methods in this study is a major strength and allowed children to engage in the study in ways preferable to them. Firstly, the use of scrapbooks, and the flexibility of the activities within them, was well received by children of all abilities. Secondly, the parent-led interviews highlight the advantages of using remote data collection techniques with populations often seen as difficult to include. Allowing children to speak with a familiar member of their family likely reduced the power dynamic which is often found in qualitative research (Punch, 2002), allowing them to open up more quickly than using researcher-red interviews. This shows that younger autistic children can take part in research and that they value having their voices and opinions heard, but that researchers may need to rethink the ways in which they design data collection tools.

One major limitation is, due to the analysis only focusing on the transcripts of parent-led interviews, that the voices of autistic children who are minimally or non-speaking are not present. It is common in research for those who do not verbalize their experiences in traditional ways to be excluded from research and more must be done to allow non-verbal and minimally verbal children to participate in research. Going forward, inclusive methods that use augmentative and alternative communication may help us better understand how autistic children experience friendships. Furthermore, the use of scrapbooks in the current study were well received by children and future studies would benefit from including the drawings of children in the analysis which would be more inclusive of those who do not communicate traditionally.

Although parent-led interviews were a strength regarding their flexibility and ease of use, they came with challenges. The researcher did not have the opportunity to probe particularly interesting and relevant responses. Furthermore, there is a chance that parents could influence children's responses, leading to bias, despite providing questions for parents to ask their children. Creating more detailed interview schedules, with clearer prompts, may be one way to overcome this challenge while retaining the approach's benefits.

Finally, the study would have benefitted from more participatory approaches so that autistic individuals could help to highlight key areas that may be of interest to the community. Future research should include the voices of autistic individuals at all stages to ensure that the research investigates areas of priority for autistic people.

## Conclusion

This study highlights the different needs and wants of autistic children, even within similar settings, and the need for better support and better education for both autistic children, their non-autistic peers and key adults. For example, including interventions that support non-autistic children in adapting their social communication to include autistic children may be beneficial. This would also reduce the pressure on autistic children to conform to social norms and in turn, increase well-being.

The paper also found that autistic children clearly have their own opinions on their friendships, with some craving large numbers of friends and others being happy on their own. It highlights the challenges older primary school autistic children may face in the lead up to school transition, and shows that autistic girls may be at risk of missing out on support due to engaging with masking, even at a young age. This shows the importance of speaking with children to give them autonomy over the support they want to receive. Findings also identified that school settings are likely to be seen as safe spaces in which children would benefit from engaging in this support. Therefore, schools may benefit from talking with children to explore if they want support aimed at friendship development prior to putting social interventions in place, as imposing unwanted friendships onto children may be detrimental to their well-being. This may be something which is especially key when supporting girls, whose social norms may indicate they want more friends than they actually want to have.

## Supplemental Material

sj-docx-1-dli-10.1177_23969415241275934 - Supplemental material for “I need them for my autism, but I don’t know why”: Exploring the friendship experiences of autistic children in UK primary schools Supplemental material, sj-docx-1-dli-10.1177_23969415241275934 for “I need them for my autism, but I don’t know why”: Exploring the friendship experiences of autistic children in UK primary schools by Laura Fox and Kathryn Asbury in Autism & Developmental Language Impairments
