# Supplementary figures and images for: “I need them for my autism, but I don’t know why”: Exploring the friendship experiences of autistic children in UK primary schools
Source: Autism Dev Lang Impair. 2024 Sep 5;9:23969415241275934. doi: 10.1177/23969415241275934 (PMC11378229; doi:10.1177/23969415241275934)

Appendix A
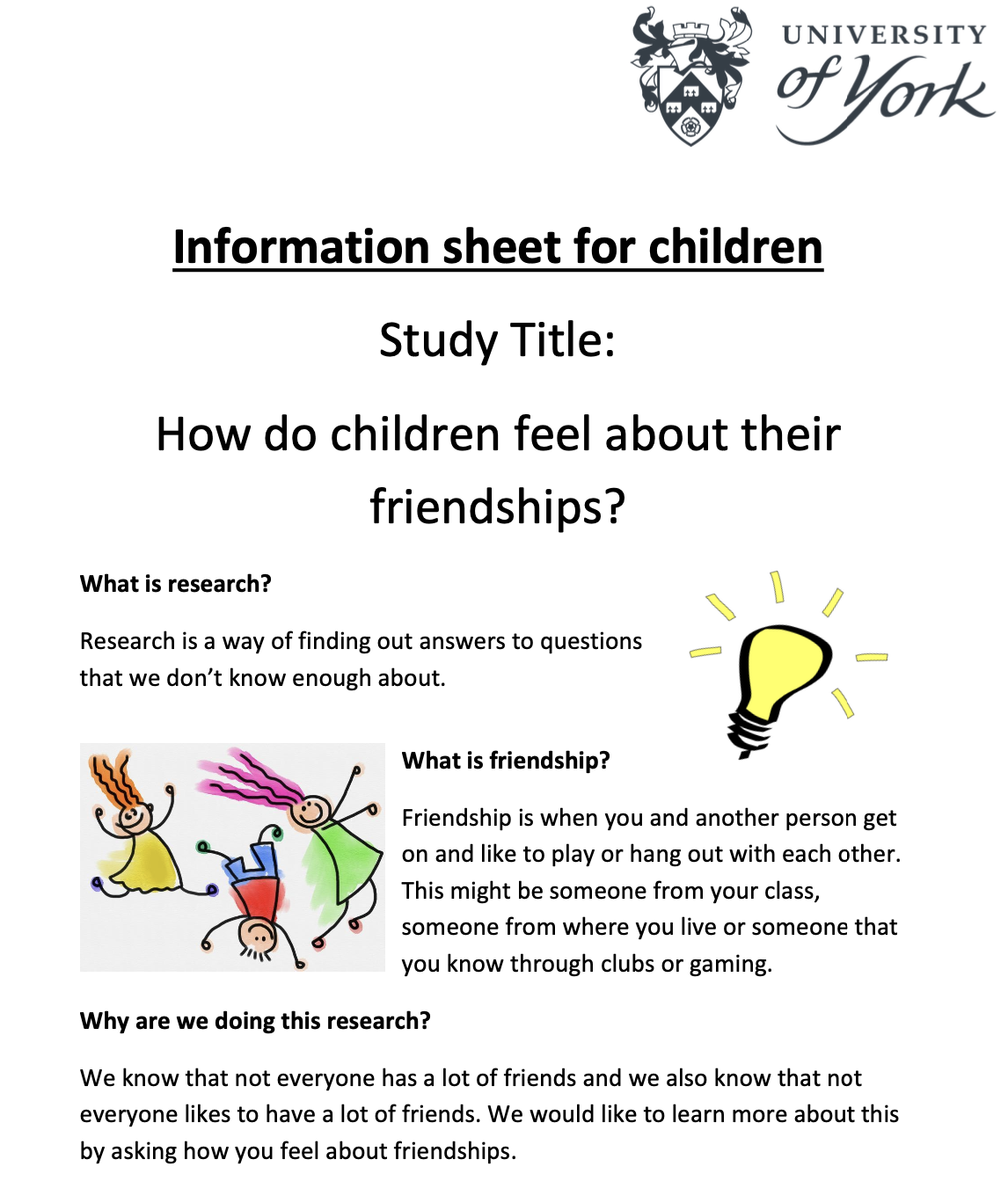


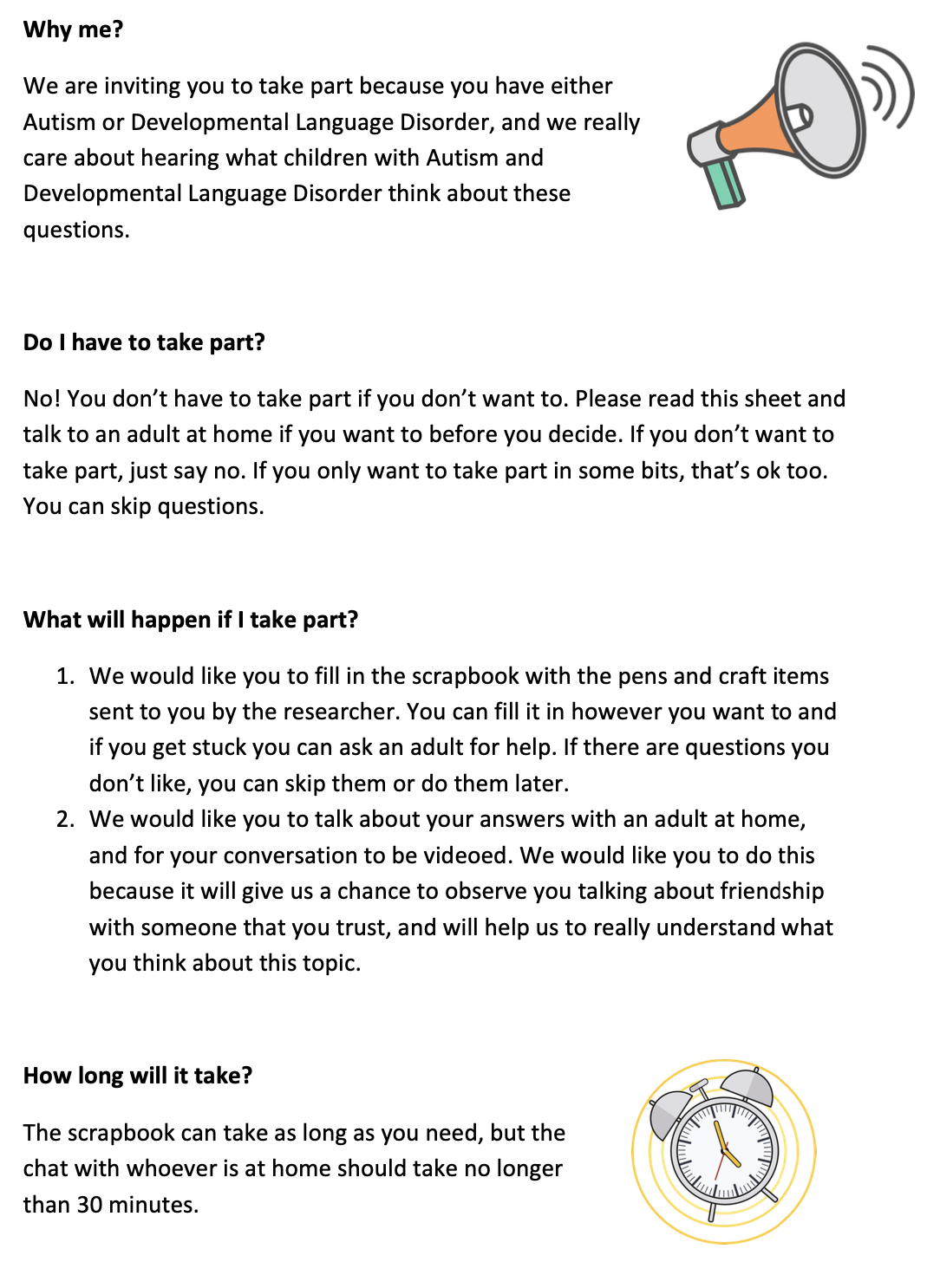

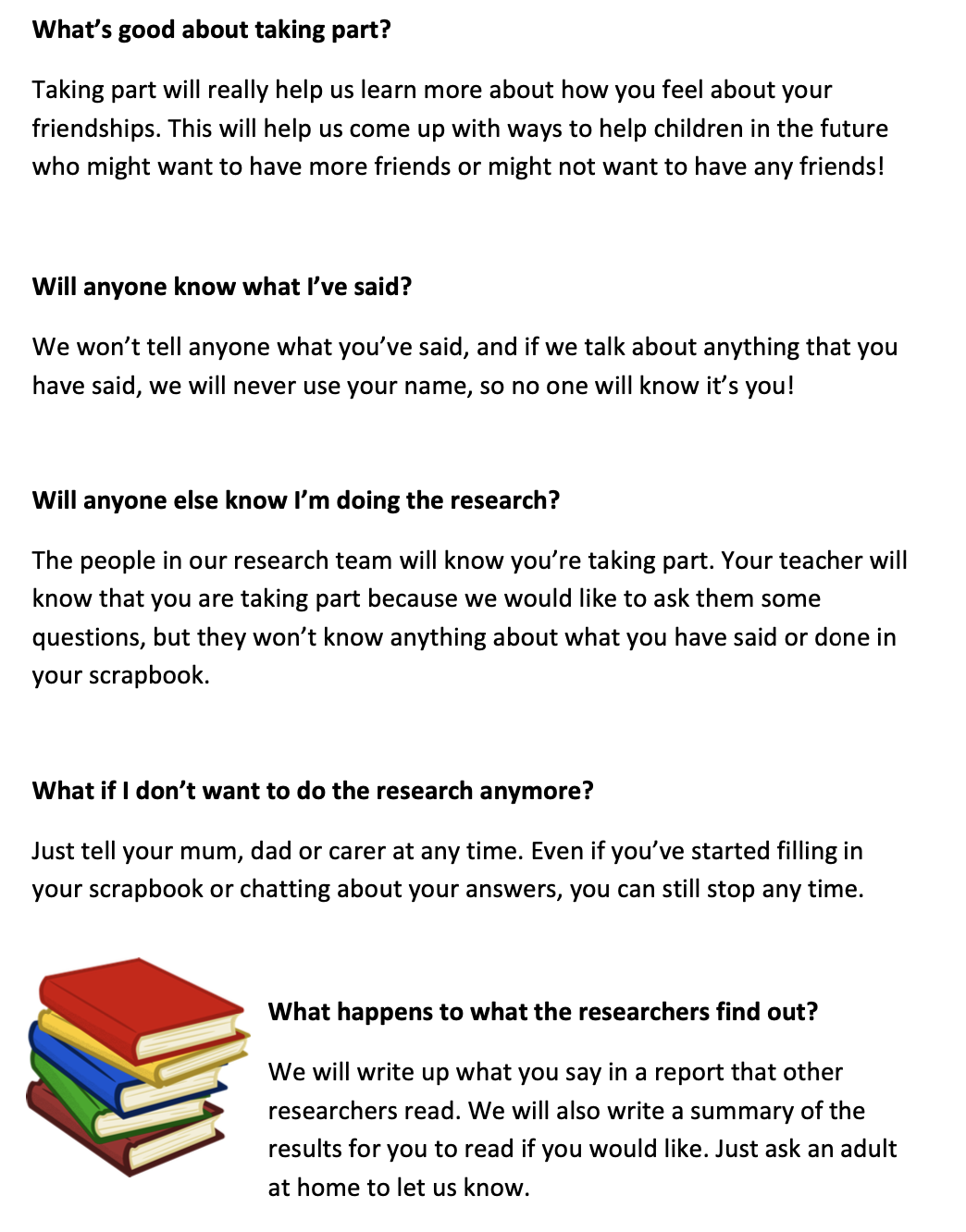

Supplement: sj-docx-1-dli-10.1177_23969415241275934 - Supplemental material for “I need them for my autism, but I don’t know why”: Exploring the friendship experiences of autistic children in UK primary schools [file sj-docx-1-dli-10.1177_23969415241275934.docx]
